# Indole‐Acetic Acid Impairs *Pseudomonas aeruginosa* Virulence and Alters Lung Infection in Mice

**DOI:** 10.1002/mbo3.70185

**Published:** 2025-12-12

**Authors:** Carlos Eduardo Dias Igídio, Camila Bernardo Brito, Rafael de Oliveira Bezerra, Samantha Neves Oliveira, Cinthia Firmo Teixeira, Bárbara Maria de Amorim‐Santos, Allanis Cristiny Oliveira Andrade, Diego Lisboa Rios, Silvia Helena Sousa Pietra Pedroso, Simone Gonçalves dos Santos, Mauro Martins Teixeira, Daniele da Glória de Souza, Camila Pacheco Silveira Martins da Mata, Caio Tavares Fagundes

**Affiliations:** ^1^ Centro de Pesquisa e Desenvolvimento de Fármacos, Instituto de Ciências Biológicas Universidade Federal de Minas Gerais Belo Horizonte Minas Gerais Brazil; ^2^ Laboratório de Interação Microrganismo‐Hospedeiro, Departamento de Microbiologia, Instituto de Ciências Biológicas Universidade Federal de Minas Gerais Belo Horizonte Minas Gerais Brazil; ^3^ Laboratório de Microbiologia Oral e de Anaeróbios, Departamento de Microbiologia, Instituto de Ciências Biológicas Universidade Federal de Minas Gerais Belo Horizonte Minas Gerais Brazil; ^4^ Hospital Risoleta Tolentino Neves Belo Horizonte Minas Gerais Brazil

**Keywords:** anti‐virulence strategies, gut‐lung axis, indoles, pneumonia, tryptophan metabolism

## Abstract

Patients in intensive care units, especially those immunocompromised, are prone to opportunistic infections, such as respiratory and urinary tract infections. Extended antibiotic use disrupts the production of microbiome‐derived metabolites, including those involved in colonization resistance to *Pseudomonas aeruginosa*, which is known for its multidrug resistance. Hence, prior antibiotic treatment has been shown to increase susceptibility to *P. aeruginosa* infection, but the role of microbiota‐derived metabolic cues in this context is still elusive. This study investigates how tryptophan metabolites from the indigenous microbiota affect *P. aeruginosa* virulence. *In vitro* tests on motility, biofilm production, and pigment quantification (pyocyanin and pyoverdine) were performed on *P. aeruginosa* strains (PAO1, PA103, PA14) and clinical isolates. Additionally, gene expression related to virulence was analyzed, and the effects of tryptophan metabolites on experimental lung infection in mice were evaluated. Indole, indoleacetic acid (IAA), and indoleacrylic acid (IA) reduced motility and pigment production. IAA and indole promoted biofilm formation, with indole having a stronger effect. Clinical isolates showed significant phenotypic diversity, and IAA was more effective at inhibiting virulence traits than indole or IA. Mice infected with bacteria grown in the presence of IAA had lower lethality and fewer polymorphonuclear leukocyte influx compared to the control group. This suggests that tryptophan metabolites, especially IAA, can modulate *P. aeruginosa* virulence and may help control infection progression.

## Graphical abstract

Screening for anti‐virulence activity exerted by tryptophan catabolites on laboratory and clinical strains of *Pseudomonas aeruginosa* showed that Indoleacetic acid (IAA) inhibits motility and pigment production, downregulates PQS gene expression, while increases oxidative stress sensitivity. These changes led to reduced inflammation and improved survival in mice infected with IAA‐exposed bacteria.

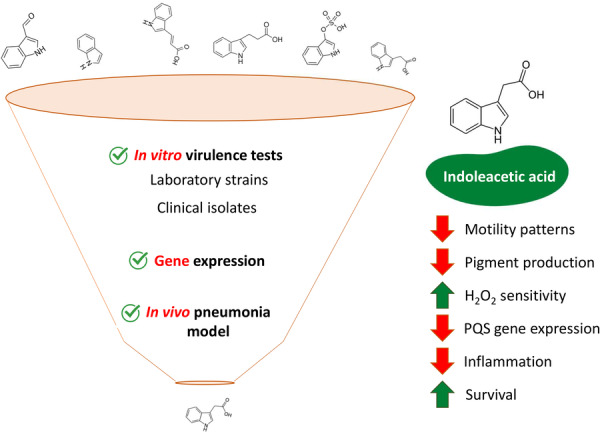

## 1 Introduction

Tryptophan (Trp) is an essential amino acid involved in protein synthesis and the production of metabolites that influence gastrointestinal, immune, and nervous system functions (Xue et al. 2023). After ingestion, Trp is mainly metabolized through the kynurenine pathway in immune and epithelial cells. It can also follow the serotonin pathway in enterochromaffin cells or undergo microbial catabolism, generating aryl hydrocarbon receptor (AHR) ligands (Modoux et al. 2021). In *Pseudomonas aeruginosa*, Trp is converted into anthranilate via the oxidative kynurenine pathway, a precursor of Pseudomonas Quinolone Signal (PQS), which regulates quorum sensing and virulence factor expression (Bortolotti et al. 2016).

*P. aeruginosa* is a Gram‐negative opportunistic pathogen responsible for acute and chronic infections, particularly in immunocompromised individuals (Qin et al. 2022). It is part of the ESKAPE + C group— *Enterococcus faecium, Staphylococcus aureus, Klebsiella pneumoniae, Acinetobacter baumannii, Pseudomonas aeruginosa, Enterobacter* spp*., and Clostridioides difficile*—which are recognized by the World Health Organization (WHO) as priority pathogens for new antimicrobial strategies (De Oliveira et al. 2020). Epidemiological studies indicate that *P. aeruginosa* accounts for 5%–14% of healthcare‐associated infections and 16–40% of ventilator‐associated pneumonia (VAP) cases (Daikos et al. 2021).

Virulence factor production enhances *P. aeruginosa* survival by evading host immune responses (Feng et al. 2021). The bacterium exhibits three motility patterns—swimming, swarming, and twitching ‐ mediated by a polar flagellum and pili, both of which contribute to adhesion, inflammation, and biofilm formation (Patankar et al. 2013). Biofilms, composed of bacterial aggregates embedded in extracellular polymeric substances (EPSs), protect against phagocytes, reactive oxygen species, and antimicrobials while facilitating nutrient exchange (Gellatly and Hancock 2013). Additionally, *P. aeruginosa* secretes pyocyanin and pyoverdine via the type II secretion system (Moradali et al. 2017). Pyocyanin induces oxidative stress, neutrophil apoptosis, and phagocytosis inhibition, while pyoverdine functions as a siderophore, sequestering host iron (Nadal Jimenez et al. 2012; Hall et al. 2016).

Prolonged antimicrobial use in intensive care units (ICUs) is a major factor in *P. aeruginosa* infections (Gomila et al. 2018). Selective pressure from antimicrobials, along with the pathogen's ability to persist on hospital surfaces, exacerbates infection severity and promotes its spread (Araújo et al. 2022; Ponce de Leon et al. 2020). Additional risk factors include co‐infections, parenteral nutrition, ICU admission, immunosuppression, mechanical ventilation, and multiple invasive devices (Alhussain et al. 2021). Studies show that 41.9% of ventilated patients with *P. aeruginosa* infections die, with inadequate antimicrobial use being a key risk factor (Timsit et al. 2017). This may be linked to both resistance development and alterations in host microbiota (dysbiosis) (Cusumano et al. 2025).

Dysbiosis impacts host metabolism, including Trp catabolism. Notably, Trp‐derived metabolites influence *P. aeruginosa* virulence (de Brito et al. 2025). Indole, a Trp‐derived metabolite, exemplifies how these compounds influence *Pseudomonas aeruginosa* virulence. It reduces the production of rhamnolipid, PQS, and pyoverdine, enhances biofilm formation, and limits lung colonization in mice (Lee et al. 2009). Mechanistically, indole modulates quorum sensing, virulence gene expression, motility, and toxin secretion. Moreover, it acts as an interspecies signaling molecule that shapes bacterial pathogenesis and host immune responses (Lee et al. 2015). Thus, antimicrobial‐induced shifts in Trp metabolism and decreased microbial‐derived indolic compounds may enhance bacterial virulence.

This study investigated the effects of Trp catabolites on *Pseudomonas aeruginosa* virulence in vitro and their impact on lung infection in mice. Our results show that Trp catabolites can alter *P. aeruginosa* virulence factors in vitro. Additionally, we showed that IAA can reduce *P. aeruginosa* virulence in vivo, leading to increased survival in mice after *P. aeruginosa* lung infection.

## 2 Results

### 2.1 Indole, IAA and IA, Intermediary Products of Tryptophan Catabolism by the Gut Microbiota, Interfere With the Virulence of *P. aeruginosa* Laboratory Strains

**Figure 1:**
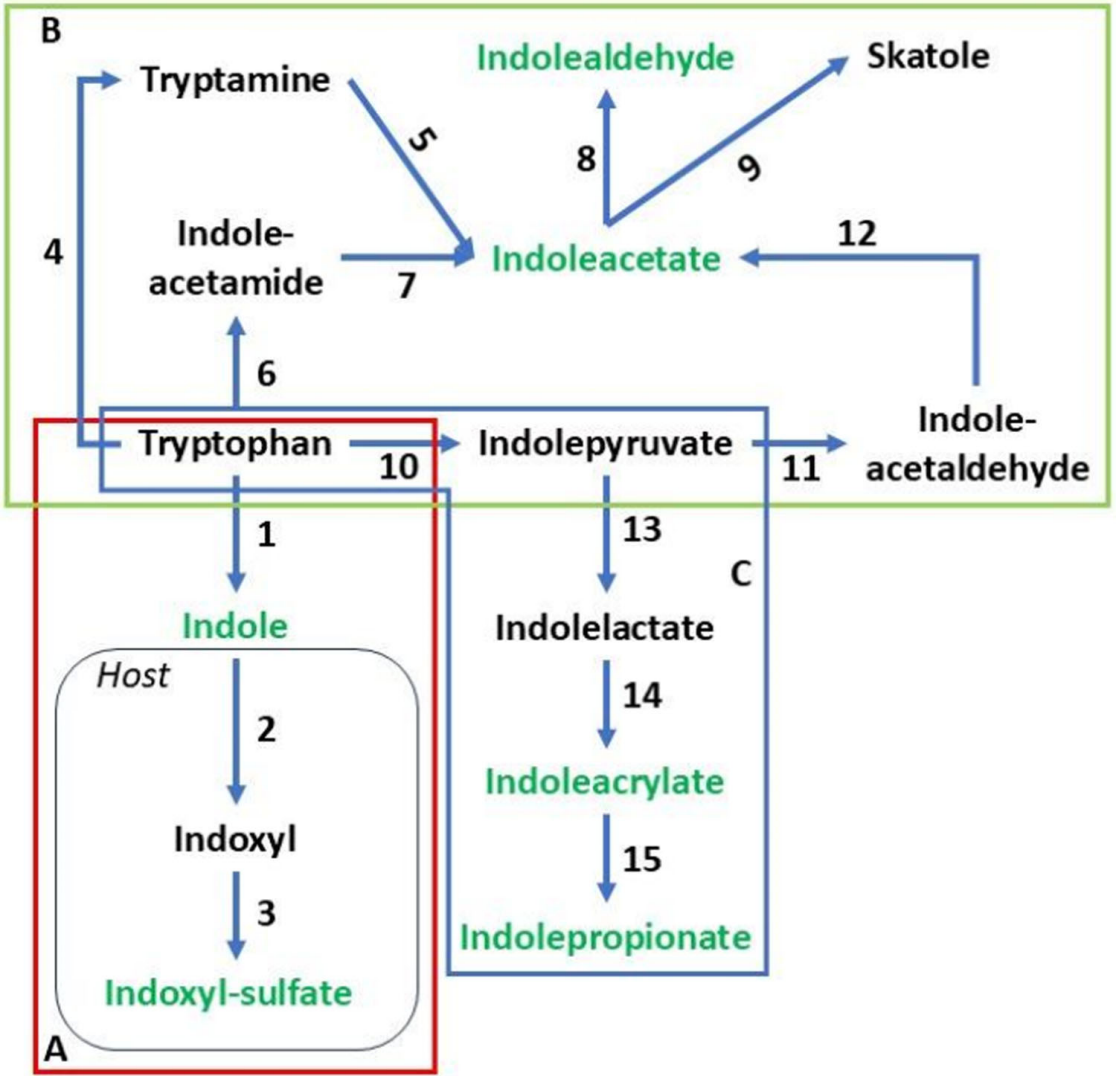
Tryptophan catabolism by bacteria. The three major pathways of tryptophan catabolism by gut microbiota, leading to end‐products that reach micromolar concentrations in the circulation are schematically represented: (A) tryptophan catabolism to indole, that is subsequently absorbed and converted to indoxyl and to indoxyl‐sulfate by the host, in the red diagram; (B) tryptophan catabolism by each of the tryptamine, indole‐acetamide, indole‐pyruvate and indole‐acetaldehyde pathways, leading to indole‐acetic acid (IAA) production which is further metabolized to indole‐carboxyaldehyde (IAld) or skatole, in the green diagram; (C) and the catabolism of tryptophan to indolepyruvate and indolelactate, leading to production of indoleacrylate (IA) and indolepropionate (IPA), in the blue diagram. The metabolites written in green were utilized in this study. The numbers refer to the enzymes involved in the catabolic process: 1 ‐ TNA: tryptophanase; 2 ‐ Cyp2E1: Cyochrome P450 family 2 subfamily E member 1; 3 ‐ SULT: sulfotransferase; 4 ‐ TrpD: tryptophan decarboxylase; 5 ‐ AmO: ammonia monooxygenase; 6 ‐ TMO: tryptophan monooxygenase; 7 ‐ IaaH: indoleacetamide hydrolase; 8 ‐ AST: aspartate aminotransferase; 9 ‐ IaaD: indoleacetaldehyde decarboxylase; 10 ‐ ArAT: aromatic aminoacid aminotransferase; 11 ‐ ID: indolepyruvate decarboxylase; 12 ‐ IaaDH: indoleacetaldehyde dehydrogenase; 13 ‐ ILDH: indolelactate dehydrogenase; 14 ‐ ILD: indolelactate dehydratase; 15 ‐ ACD: acetyl‐CoA dehydrogenase.

Microbial tryptophan catabolism in the gut involves diverse pathways, as shown in Figure 1. Briefly, there are three major pathways of tryptophan catabolism by gut microbiota, leading to end‐products that reach micromolar concentrations in the circulation: tryptophan catabolism to indole, that is subsequently absorbed and further modified to indoxyl‐sulfate by the host (Figure 1A); tryptophan catabolism by different pathways, leading to IAA and IAld production (Figure 1B); and the catabolism of tryptophan to indolepyruvate and indolelactate, leading to production of IA and IPA (Figure 1C). We aimed to evaluate the potential anti‐virulence effect exerted by these major gut microbiota‐derived tryptophan end‐products in two different laboratory strains of *Pseudomonas aeruginosa*, PAO1 and PA14.

**Figure 2:**
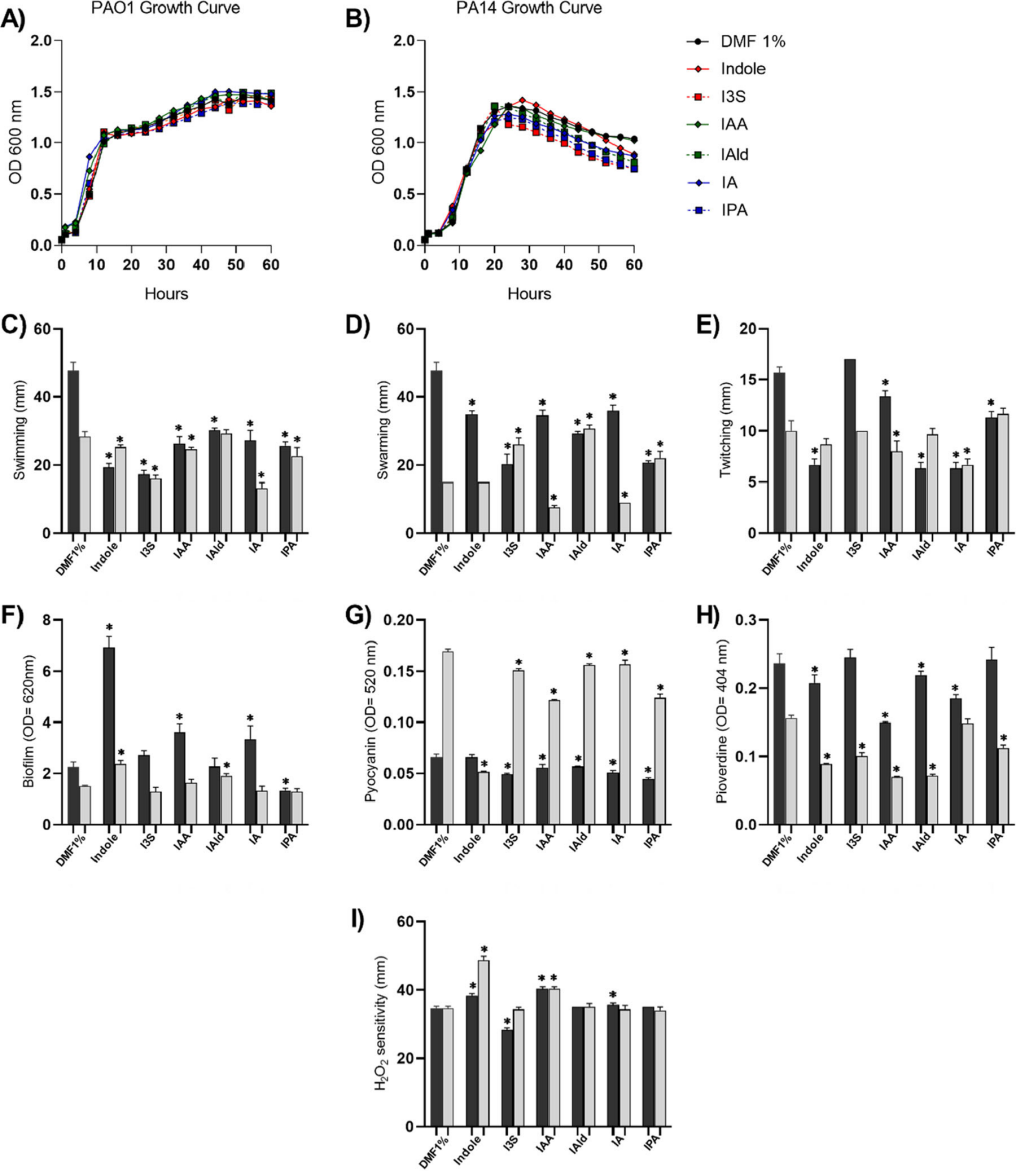
Effect of tryptophan metabolites on the growth and the virulence of *P. aeruginosa*. (A,B) 20 µL of the inoculum at 10^6^ CFU/mL of PAO1 (A) or PA14 (B) was added to 180 µL of LB broth containing DMF or the different tryptophan catabolites (at 100 µM) in a 96‐well plate. The plate was incubated at 37°C in a plate reader for 60 h. Every 1 h, the absorbance was read at 600 nm to create an absorbance versus time curve. Results are shown as mean from three replicates for each treatment. (C–I) PAO1 (dark gray bars) or PA14 (light gray bars) were evaluated for the virulence phenotypes after exposure to indole, I3S, IAA, IAld, IA, and IPA metabolites (100 µM). (C) Swimming motility, expressed as mm of motility halo; (D) Swarming motility, expressed as mm of motility halo; (E) Twitching motility, expressed as mm of motility halo; (F) Biofilm matrix production, as expressed as O.D. of crystal violet dye incorporation; (G) Pyocyanin production, expressed as O.D. in the extract from culture supernatants; (H) Pyoverdine production, expressed as O.D. in the extract from culture supernatants; (I) Hydrogen peroxide sensitivity, expressed as mm of inhibition halo. N = 3 per treatment. *P < 0.05 when compared to DMF‐exposed inocula.

First, we evaluated whether these catabolites interfered in bacterial planktonic growth. Tryptophan metabolites, at 100 µM, did not change the growth curve of PAO1 (Figure 2A) and PA14 (Figure 2B) strains. The PAO1 strain reached the stationary phase approximately 12 h after incubation, while the PA14 strain reached the stationary phase later, around 18 h after incubation. Furthermore, the peak optical density between the DMF1%‐exposed control group and the groups grown in the presence of tryptophan metabolites were similar (Figure 2).

**Figure 3:**
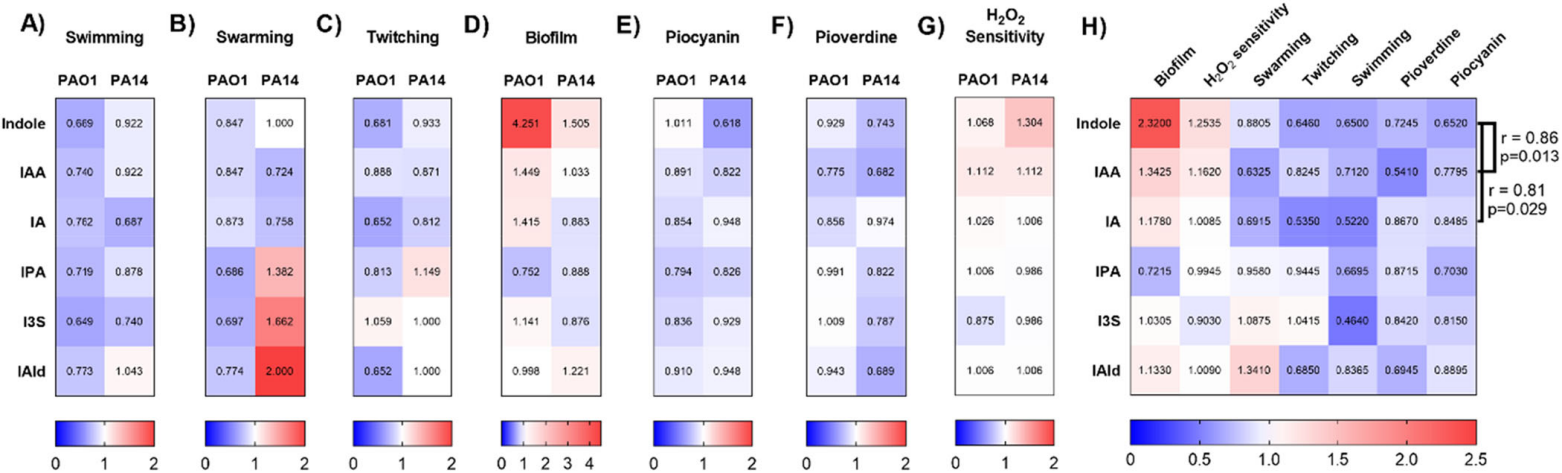
Effect of tryptophan metabolites on parameters related to virulence of *P. aeruginosa* and correlation analysis between them. (A–G). The effect of each metabolite on the PAO1 and PA14 strains was represented as a heatmap for each of the analyzed parameters. The average effect of each metabolite on the two strains was calculated obtaining the relative change over DMF‐exposed controls. (H) Heatmap showing the average effect for each metabolite on the two strains analyzed in A–G. Effects obtained for indole on the different parameters showed a positive correlation to the effect exerted by exposure to IAA or IA. All values were normalized to 1. Values above > 1 represent an increase, =1 no effect or < 1 decrease of the analyzed parameter. N = 3 per group.

Subsequently, we conducted several tests assessing the effect of these metabolites on *P. aeruginosa‐*associated virulence phenotypes, including motility patterns, biofilm production, pigment release and resistance to oxidative stress. Tryptophan metabolites at 100 µM were able to alter parameters associated with *P. aeruginosa* virulence. Exposure to any of the metabolites reduced *P. aeruginosa* swimming, except for IAld, which did not interfere with this motility pattern of the PA14 strain (Figures 2C and 3A). A similar profile was found regarding swarming motility pattern. All the metabolites reduced swarming halo formation by the PAO1 strain. The picture was more heterogenous when analyzing the effects on swimming by the PA14 strain: while exposure to indole had no effect, exposure to IAA or IA reduced swimming halos, while exposure to I3S, IAld or IPA increased them (Figures 2D and 3B). Only IAA and IA reduced twitching motility in both *P. aeruginosa* strains, while indole, IAld and IPA reduced this motility pattern on the PAO1 strain (Figures 2E and 3C). Therefore, all the metabolites interfered in *P. aeruginosa* motility patterns, except for I3S, that had no effect in twitching motility in none of the tested strains.

Exposure to indole led to increased biofilm formation in both strains, with a prominent effect in the PAO1 strain, as previously published (Lee et al. 2009). In addition, IAA and IA led to slight increases in biofilm formation by PAO1 strain and IAld led to greater biofilm deposition by PA14 strain. On the contrary, exposure to IPA had a negative, yet small, effect in biofilm formation by PAO1 (Figures 2F and 3D), suggesting that these metabolites had minor effects in the sessile lifestyle of *P. aeruginosa*. The production of pyocyanin and pyoverdine pigments was also impaired by the action of tryptophan metabolites. In general, all the metabolites reduced pyocyanin production by both strains, except for indole, that did not affect pigment production by PAO1 strain, despite a robust effect on the PA14 strain (Figures 2G and 3E). As the output of pyocyanin by the PA14 strain is higher when compared to the PAO1 strain, thus the effect of the metabolites in reducing the production of this pigment was more pronounced in PA14. Only I3S and IPA had no effect on pyoverdine production by the PAO1 strain, while exposure to IA had no impact on the production of this siderophore by the PA14 strain (Figures 2H and 3F). Finally, sensitivity to hydrogen peroxide was evaluated by the agar diffusion technique. Exposure to indole or IAA increased the sensitivity to hydrogen peroxide of both strains. Incubation with IA also increased the inhibition halo in the PAO1 strain. Interestingly, I3S made PAO1 more resistant to the effect of H_2_O_2_ (Figures 2I and 3G).

The effects of tryptophan metabolites on factors associated with the virulence of PAO1 and PA14 strains were also presented as heatmaps, with relative effects shown (Figure 3A–G). This type of analysis allowed us to compare the effects of each metabolite on the several parameters on both strains. Correlation analysis between the effect of these metabolites for the various parameters analyzed showed that there is a strong positive correlation between the effects exerted by the exposure to indole or IAA and exposure to indole or IA, suggesting that response to these metabolites could involve similar mechanisms (Figure 3H). Because these three tryptophan catabolites inhibited all the virulence parameters assessed (except for biofilm deposition) in both strains of *P. aeruginosa*, they were selected for the following experiments.

### 2.2 Characterization of the Virulence Profile of *P. aeruginosa* Clinical Isolates

**Figure 4:**
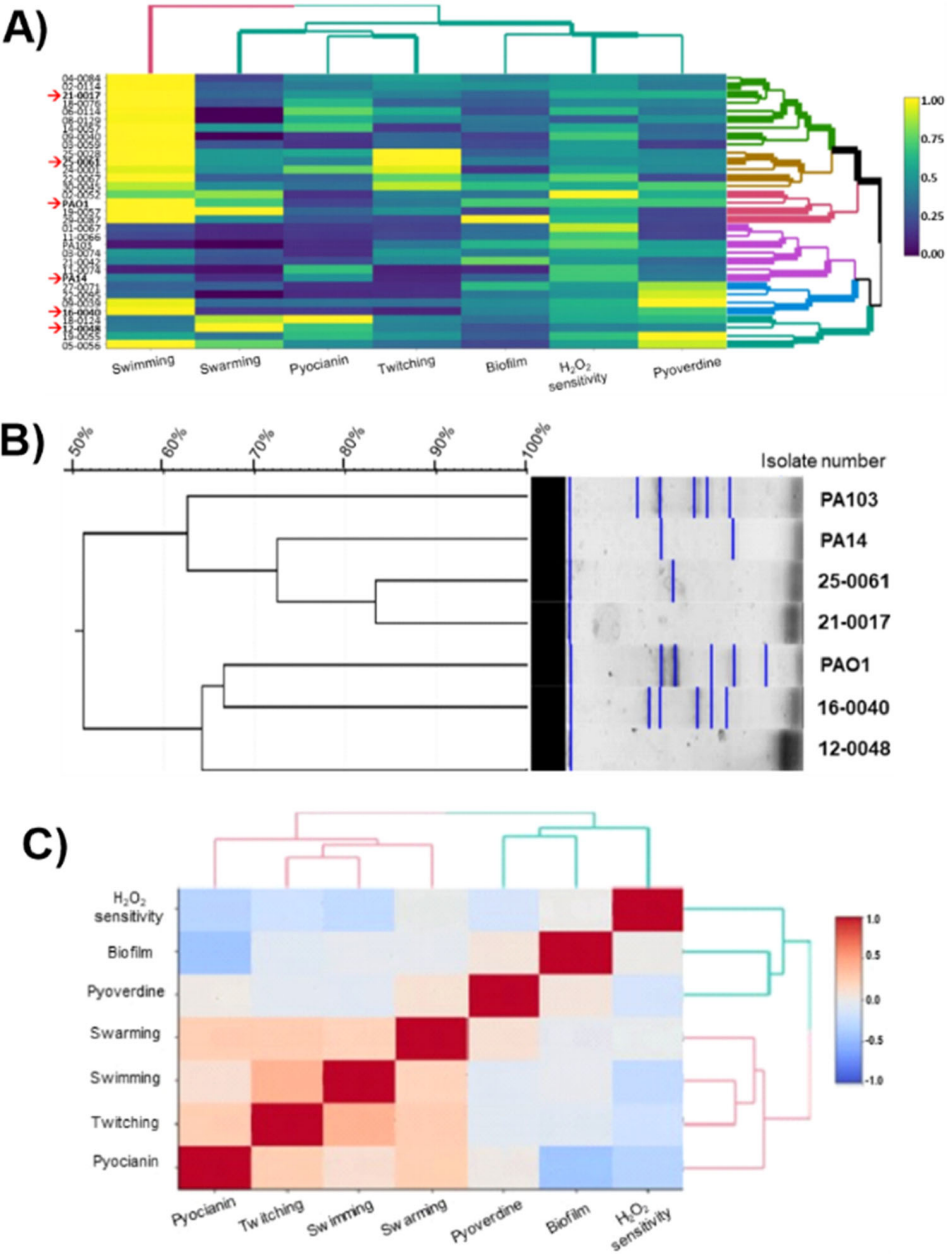
Phenotypic and genotypic analysis and clustering of clinical isolates of *P. aeruginosa*. (A) The heat map shows the average of the fold change over the overall mean, calculated between the 30 strains tested shown in Supplementary figure 1 (N = 3 per strain, for each parameter). The clinical isolates and reference strains of *P. aeruginosa* were distributed into six groups according to the virulence profile for each of the parameters analyzed. The isolate that came closest to the average values within each cluster and the reference strains (marked with a red arrow) were selected for the following analysis. (B). Dendogram calculated using the segregation pattern for ERIC‐PCR amplicons for the strains selected in Figure 4A; (C) Heatmap showing correlation analysis for virulence. The heatmap shows two clusters that grouped pyoverdine, biofilm and sensitivity to H_2_O_2_ (blue) and pyocyanin production, swimming, swarming and twitching motilities (pink).

*P. aeruginosa* presents great genetic variability, leading to major phenotypic differences between different strains, which impacts their virulence profile. Therefore, any study conducted in laboratory strains represents a limitation to the scenario found in clinical settings. Then, our next step was to evaluate whether the selected tryptophan catabolites exerted similar anti‐virulence effects on *P. aeruginosa* strains isolated from clinical specimens. To this end, first we assessed the virulence profile of 30 clinical isolates of *P. aeruginosa* obtained from samples of the Intensive Care Unit (ICU) of a large teaching hospital, in Belo Horizonte, Brazil. The same virulence parameters evaluated in the previous section were assessed for these 30 strains. As demonstrated in Figure S1, there was a large phenotypic variation among the clinical isolates of *P. aeruginosa* for any of the parameters assessed. These measurements were normalized, using the average of the values obtained for the 30 clinical samples and three laboratory strains (PAO1, PA14 and PA103) and a correlation analysis allowed to group each strain into six clusters that reflect their virulence profile, as depicted in Figure 4A. Then, the *P. aeruginosa* isolate that was closest to the median value for each parameter among the samples from its respective cluster was selected for the next series of experiments. *P. aeruginosa* clinical isolates 21‐0017 (green cluster), 25‐0061 (yellow cluster), 16‐0040 (blue cluster) and 12‐0048 (gray cluster) as well as PAO1 (pink cluster), PA14 and PA103 (purple cluster) strains were then subjected to molecular profiling by ERIC‐PCR (Figure 4B). Through the detection of ERIC sequences, the selected clinical isolates showed a minimum similarity of 62%, reflecting a great variability between the strains. 21‐0017 and 25‐0061 samples showed closer similarity in ERIC sequence profiles (84%). As expected from the results obtained in the phenotypic analysis and from previous reports, PA14 and PA103 clustered together, in a separate branch from PAO1. However, in contrast to what was found in the phenotypic analysis, 16‐0040 and 12‐0048 samples were genetically closer to PAO1 than to PA14, while 21‐0017 and 25‐0061 samples were genetically closer to PA14 than to PAO1 (Figure 4B). In conclusion, this phenotypic and molecular characterization allowed us to select a diverse array of *P. aeruginosa* strains to be used in the following experiments.

Also, the phenotypic characterization of clinical isolates allowed us to conduct a correlation analysis seeking for association between each of the virulence parameters assessed. The heatmap shown in Figure 4C shows that there is a positive correlation between the motility patterns and pyocyanin production, as clinical isolates with greater motility exhibited greater production of this pigment. Also, pyoverdine is positively associated to biofilm formation, while sensitivity to H_2_O_2_ is negatively associated to these two parameters. Therefore, clinical isolates that make less biofilm, produce less pyoverdine and are more sensitive to hydrogen peroxide. Because of these association found between virulence parameters, we limited our next experiments to swarming, pyocyanin, pyoverdine and sensitivity to H_2_O_2_ assays.

### 2.3 IAA Is More Effective in Inhibiting the Virulence Phenotypic Traits of *P. aeruginosa* Clinical Isolates Than Indole or IA

**Figure 5:**
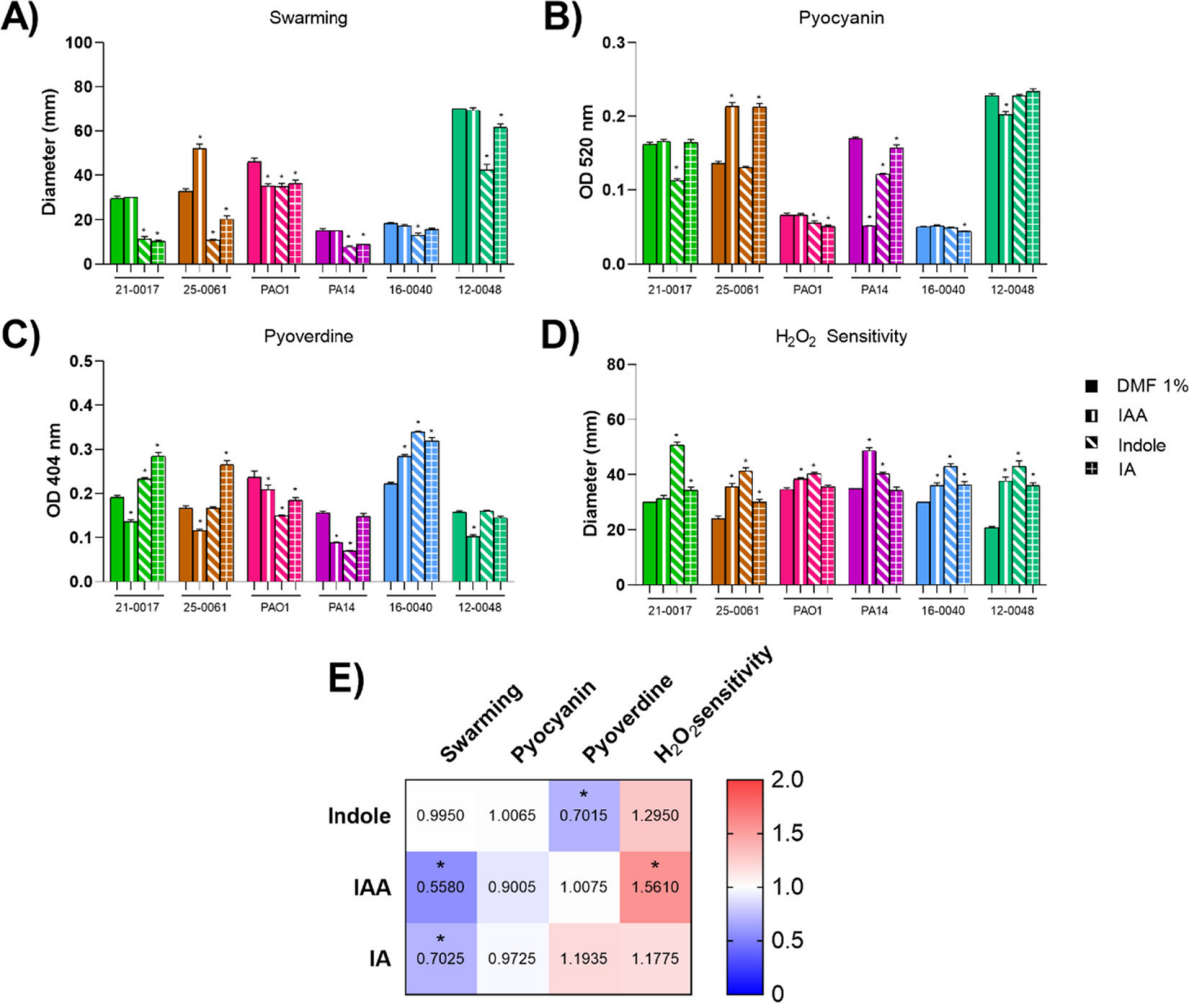
IAA, Indole, and IA effects on swarming motility, pyocyanin and pyoverdine production and sensitivity to H_2_O_2_ in clinical isolates of *P. aeruginosa*. Selected *P. aeruginosa* clinical isolates were evaluated for the virulence phenotypes after exposure to indole, IAA and IA metabolites (100 µM). (A) Swarming motility, expressed as mm of motility halo; (B) Pyocyanin production, expressed as O.D. in the extract from culture supernatants; (C) Pyoverdine production, expressed as O.D. in the extract from culture supernatants; (D) Hydrogen peroxide sensitivity, expressed as mm of inhibition halo; The average for the values obtained in A–D for the six strains was represented using the heatmap (E). Clinical isolates were represented on the graph according to the color defined by the phenotypic grouping (Figure 4A). N = 3 per strain. *P < 0.05 when compared with DMF‐exposed bacteria.

The effect of exposure to indole, IAA or IA on swarming motility, pyocyanin and pyoverdine production and sensitivity to hydrogen peroxide by the selected *P. aeruginosa* samples and strains is depicted in Figure 5. Overall, indole exposure did not reduce swarming motility in clinical isolates, as it did in laboratory strains. On the contrary, it increased swarming motility in 25‐0061 isolate. However, IAA and IA reduced swarming halos in the four clinical isolates tested, except for the 16‐0040, that was not responsive to IA (Figure 5A). As for pyocyanin production, indole and IAA reduced the pigment in isolates 12‐0048 and 21‐0017. On the other hand, production was higher in isolate 25‐0061 when grown in the presence of indole and IA (Figure 5B). Incubation with indole reduced pyoverdine production in three out of four clinical isolates, in addition to the laboratory strains. On the contrary, exposure to indole led to increased pyoverdine output by 16‐0040 isolate. IAA exposure promoted pyoverdine production in 21‐0017 and 16‐0040 isolates, and incubation with IA promoted siderophore production in all the clinical isolates, except for the 12‐0048 sample (Figure 5C). In general, the effects exerted by the metabolites are discrete in isolates with low expression of the pigments and did not follow the trend found in laboratory strains. Finally, the H_2_O_2_ sensitivity profile observed for the PAO1 strain was also similar for the clinical isolates. Indole, IAA, and IA were able to make most of the *P. aeruginosa* clinical isolates more sensitive to reactive oxygen species, except for the isolate 21‐0017, that was not responsive to incubation with indole (Figure 5D).

Figure 5E shows a heatmap with the average of the relative effect exerted by incubation with each of the three metabolites on the four parameters assessed in the six *P. aeruginosa* samples. There was no correlation in response to the metabolites between the six strains. In conclusion, while Indole exposure reduced average pyoverdine production, IA exposure reduced the mean swarming halo. IAA was the only metabolite to affect two virulence parameters in the several strains, inhibiting swarming motility and resistance to H_2_O_2_, yet not affecting pigment production (Figure 5E).

### 2.4 Exposure to IAA, but Not to Indole or IA, Changes the Course of *P. aeruginosa* Lung Infection in Mice

**Figure 6:**
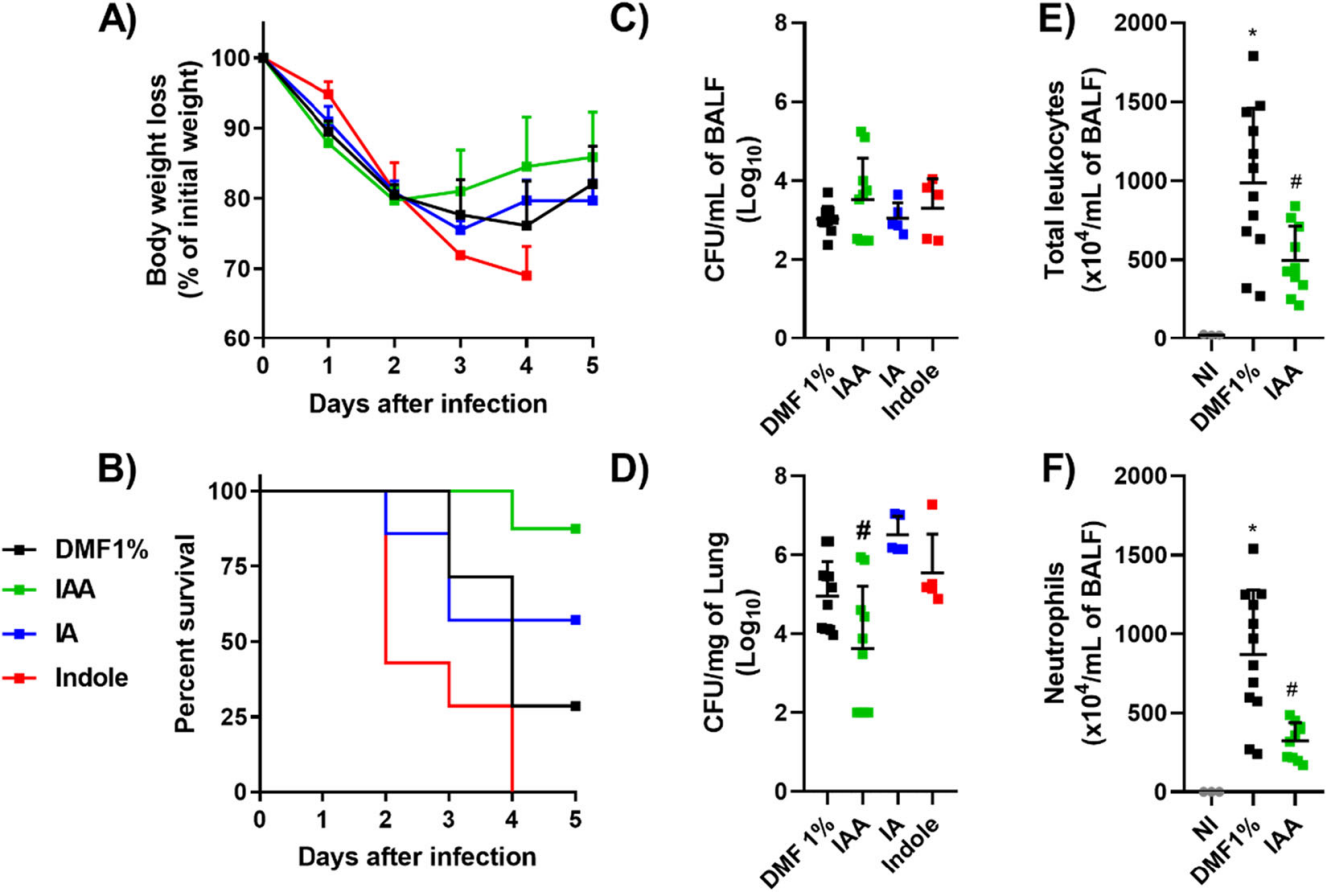
IAA exposure results in reduced *P. aeruginosa* virulence in a murine model of lung infection. PAO1 strain was grown in the presence of DMF, indole, IAA or IA and then inoculated into C57BL6j by i.n. route (A,B) Inoculated mice were monitored for body weight loss, expressed as percentual of initial weight (A) and percentual survival rates (B); (C–F). Inoculated mice were anesthetized and euthanized 24 h after inoculation for BALF and lung harvest for evaluating bacterial load in bronchoalveolar lavage (C) and lungs (D), expressed as Log_10_ CFU/mL and number of total leukocytes (E) and neutrophils (F) in BALF, expressed as number of leukocytes per mL of BALF. N = 5‐9 per mouse group. *P < 0.05 over NI group. # *P* < 0.05 over group infected DMF‐exposed bacteria.

Our next step was to assess the effects of exposure to tryptophan catabolites on the virulence of PAO1 strain *in vivo*. PAO1 strain grown in the presence of indole, IAA IA, or the vehicle DMF 1% for 24 h were inoculated in mice intranasally, and mice were monitored for 5 days. *P. aeruginosa* lung infection led to body weight loss, irrespective of the growth conditions (Figure 6A). However, mice infected with PAO1 grown in the presence of the IAA showed a higher percentage of survival after 5 days of infection. The group infected with IAA‐exposed bacteria had approximately 85% survival while only 25% of the group infected with vehicle‐exposed bacteria were alive on the 5th day post infection (*p* = 0.0195). Also, mice infected with indole‐ or IA‐exposed bacteria succumbed to infection at similar rates to those found in the control group (Figure 6B).

Another experiment was conducted to evaluate bacterial loads in the alveolar space and in the lung parenchyma of infected mice. Despite no differences in bacterial load recovered from the bronchoalveolar lavage fluid from the different groups of infected mice after 24 h of infection (Figure 6C), it was observed that mice infected with the PAO1 strain grown in the presence of IAA had a lower bacterial load in the lung tissue when compared to mice infected with the bacteria grown in the presence of the DMF1% vehicle (Figure 6D). Interestingly, mice from the IAA group had a lower influx of leukocytes into the alveolar space (Figure 6E) with reduced neutrophil counts compared to the DMF1% group (Figure 6F).

**Figure 7:**
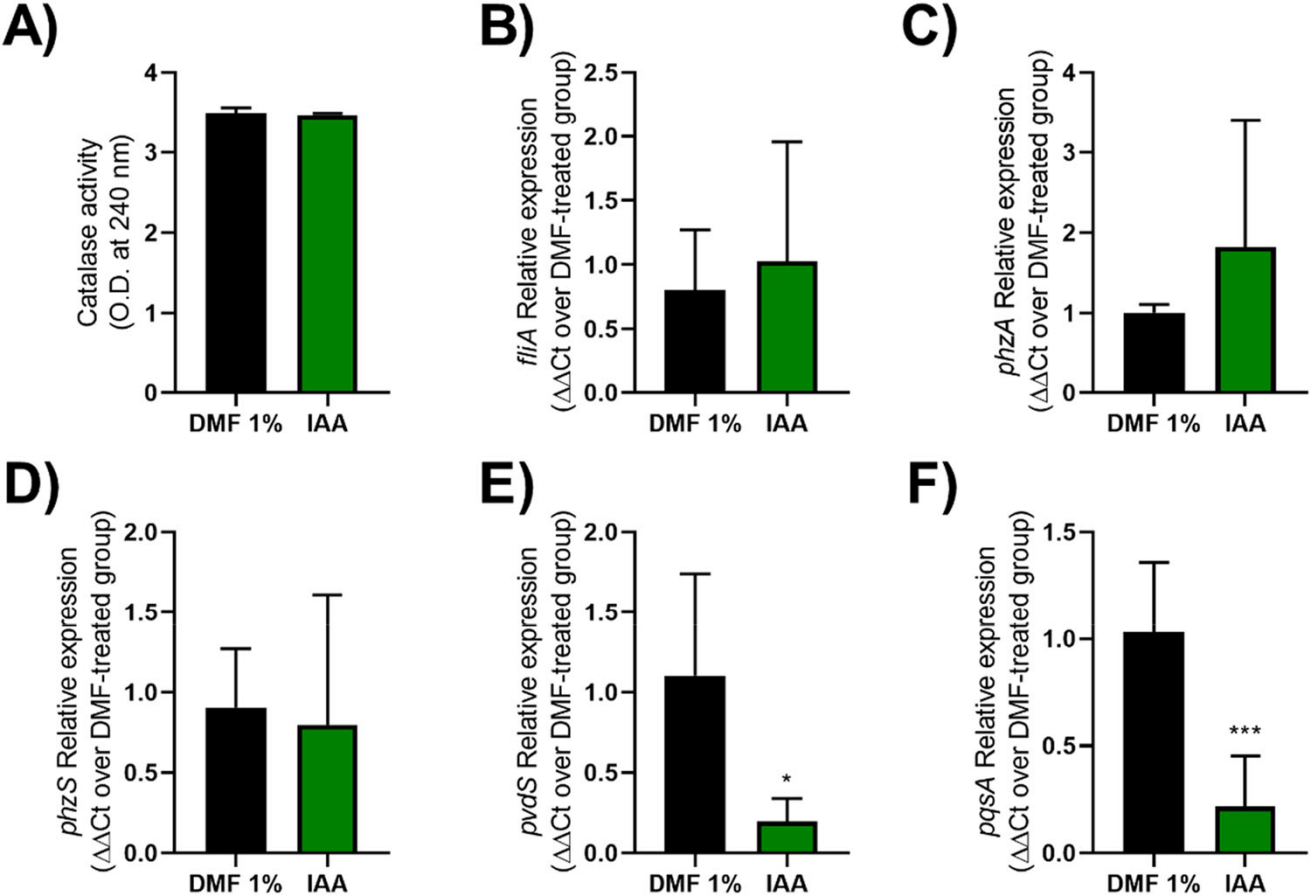
Effect of IAA on catalase activity and on the expression of genes related to virulence of *P. aeruginosa*. (A) Samples of PAO1 grown in the presence or absence of IAA for 16 h were subjected to sonication and the extracts were assayed for hydrogen peroxide degradation activity. Results are shown as O.D. at 240 nm. (B–F) Samples of PAO1 grown in the presence or absence of IAA for 8 h were subjected to RNA extraction for subsequent gene expression analysis by RT‐qPCR for *fliA* (B), *phzA* (C), *phzS* (D), *pvdS* (E), and *pqsA* (F). The results were expressed as the ΔΔCT value. *P < 0,05. N = 4 per incubation condition.

As IAA had a more pronounced effect on the virulence of *P. aeruginosa* strains, both *in vitro* and *in vivo*, the next experiments were conducted only in IAA‐exposed bacteria. First, we evaluated the hydrogen peroxide‐degrading activity in protein extracts obtained from *P. aeruginosa* cultures in the absence or presence of the metabolite. There was no significant difference in the reduction of O.D. at 240 nm between the two groups (Figure 7A). This indicates that the effect of IAA on sensitivity to H_2_O_2_ in *P. aeruginosa* does not occur directly on the enzymatic activity. Subsequently, the effect of IAA on the expression of five quorum sensing‐dependent genes associated with the virulence phenotypes assessed in this study were investigated. Incubation with IAA at 100 µM did not interfere in *fliA, phzA or phzS* expression (Figure 7B‐D), involved in flagellar‐mediated motility and pyocyanin production, respectively. However, IAA exposure led to reduced expression of *pvdS* gene (Figure 7E), gene involved in pyoverdine production. Also, IAA exposure led to reduced *pqsA* expression (Figure 7F). In conclusion, the anti‐virulence effects exerted by IAA in *P. aeruginosa* involve the modulation of virulence factor gene expression.

## 3 Discussion

*Pseudomonas aeruginosa* is a genetically complex microorganism that harbors multiple virulence factors (Subedi et al. 2018). This study evaluated the impact of tryptophan catabolites from the indigenous microbiota on *P. aeruginosa* virulence. Tryptophan metabolism plays a key role in immunological homeostasis and inflammatory responses (Le Floc'h et al. 2011), and its disruption may contribute to metabolic and infectious diseases (Sitkin et al. 2017). Certain gut bacteria, such as *Escherichia coli*, use indole to compete with *P. aeruginosa*, reducing its siderophore production (pyoverdine and pyochelin) and other virulence factors (Chu et al. 2012). Additionally, *P. aeruginosa* may utilize indole to enhance biofilm formation in the intestinal tract, evading host defenses by regulating virulence factors until reaching a sufficient cell density (Williams et al. 2007). Indole functions as an interspecies signaling molecule with roles in bacterial pathogenesis and eukaryotic immunity (Lee et al. 2015), yet the effects of other tryptophan catabolites on *P. aeruginosa* virulence remain poorly understood. *P. aeruginosa* does not produce indole, but it is able to degrade the metabolite over time. Lee et al. demonstrated that after 7 h of incubation of *P. aeruginosa* in a culture medium containing indole, the concentration of the metabolite reduced from 1 to 0.68 mM (Lee et al. 2009). The tryptophan metabolites tested in this project did not cause a decrease in the growth of the PAO1 strain, however it was noted that the PAO1 grown in the presence of the metabolites reaches the stationary phase more quickly, which may indicate that *P. aeruginosa* uses the degradation of the indole ring as a source of carbon and energy to accelerate their growth in a concentration‐independent effect. According to Lelong et al. indole induces a premature expression of the Crl regulatory protein, which increases the activity of the RNA polymerase RpoS subunit that controls the expression of several genes involved in the stationary phase and in response to different stress conditions (Lelong et al. 2007).

The prominent effect of indole and indoleacetic acid on biofilm formation and motility of *P. aeruginosa* indicates that these metabolites may be involved in the transition from the mobile state to the sessile state in *P. aeruginosa*. Bacterial biofilm formation is often associated with reduced metabolism, pili and flagellum activity, and virulence. It is known that bacterial strains from patients with chronic infections, for example, are commonly characterized by the inactivation of the MucA gene, which encodes a negative transcriptional regulator that represses bacterial stress pathways. This results in a mucoid conversion characterized by increased production of the extracellular alginate polysaccharide that makes up the biofilm. Furthermore, MucA inactivation promotes a stress response that represses bacterial metabolism, motility, and virulence (Folkesson et al. 2012). Therefore, the anti‐virulence effect exerted by indole, IAA or IA in the *P. aeruginosa* strains tested may involve transition to the sessile lifestyle.

*P. aeruginosa* can adapt to the adverse environment in hosts by secreting a variety of virulence factors, which contribute to successful infection and causing disease (Vidaillac and Chotirmall 2021). The regulation of these virulence factors is coordinated by releasing autoinducing molecules of Quorum Sensing (QS) (e.g., Las, Rhl, Pqs, and Iqs) that are dependent on cell density. Thus, QS assists in the adaptation of large populations and survival to hostile environments and, consequently, in the establishment of the infectious process (Yang et al. 2021). Therefore, the QS system has become an important target for the development of new antimicrobial therapies (Qin et al. 2022). Most of the factors associated with the virulence of *P. aeruginosa* are regulated by the quorum sensor through the autoinducer molecules 3O‐C12‐HSL and C4‐HSL and PQS, which arouses interest in the search for anti‐quorum agents (Miranda et al. 2022). IAA seems to reduce the expression of pqsA, a gene that catalyzes the formation of antraniloil‐CoA, which is the initial stage for entry into the biosynthetic pathway of the PQS autoinducer. This indicates that this metabolite can interfere with the virulence of *P. aeruginosa* via the PQS system.

In addition to coordinating the expression of pathogenicity factors, the PQS system also acts in the response to host oxidative stress. According to Haussler and collaborators, PQS‐producing strains (mutant to pqsA or pqsH) exhibit increased resistance to H_2_O_2_ compared to the wild type strains. These results demonstrate that 4‐quinolone PQS sensitizes bacteria to oxidative stress (Häussler and Becker 2008). Therefore, IAA effect on the resistance to H_2_O_2_ by *P. aeruginosa* might involve other mechanisms than the control of PQS production or catalase activity.

Population genomics studies reveal extensive genetic diversity within the clinical isolates of *P. aeruginosa*, including the coexistence of highly divergent lineages acquired by patient‐to‐patient transmission (Kümmerli et al. 2015). The clinical consequences are not fully understood, however, given the extensive phenotypic diversity, there are clear implications for false diagnoses (Winstanley et al. 2016). In this context, the study in clinical isolates is necessary due to their genotypic and phenotypic diversity, so expanding the research beyond laboratory strains is important for understanding the mechanisms of pathogenicity of *P. aeruginosa*.

The diversified effect of metabolites in clinical isolates suggests that the way of grouping the samples still needs to be improved, both with the evaluation of other virulence parameters and new forms of classification. In this sense, it is important to perform more detailed genetic analysis, as well as to know the profile of patients from whom such bacteria were isolated. Together, these data will contribute to understanding virulence dynamics of *P. aeruginosa* strains, deciphering the determinants of response to tryptophan catabolites and, consequently new strategies for the development of anti‐virulence therapies. Regardless of that, our results show that IAA inhibits virulence phenotypic traits also in *P. aeruginosa* clinical isolates, confirming its potential as an anti‐virulence metabolite.

The effect of IAA on the virulence of *P. aeruginosa* was especially prominent during lung infection in mice. In this model, we highlight the reduction of bacterial load in the lung of animals that received PAO1 grown in the presence of IAA. In addition, it was observed a lower influx of leukocytes, especially polymorphonuclear cells, into the bronchoalveolar space of mice infected with the IAA‐exposed bacteria. Neutrophils are fundamental parts for the elimination of *P. aeruginosa*, since, at the infectious site, they will release bactericidal substances such as serine proteases and nitric oxide in addition to the elimination of the bacterium via phagocytosis (Gonçalves‐de‐Albuquerque et al. 2016). However, it is important to emphasize that, although necessary for the control of bacterial load, the recruitment of neutrophils is closely related to the tissue lesion at the inflammatory site (Wang et al. 2019). We believe that the reduction in inflammatory activity secondary to diminished bacterial invasion into lung tissue may have contributed to lower tissue damage and, consequently, increased survival of animals infected with PAO1 grown in the presence of this metabolite.

In conclusion, our data suggest that metabolites from tryptophan metabolism interfere with the virulence profile of *Pseudomonas aeruginosa*. In addition, the present study demonstrated the enormous variety of virulence phenotypes in clinical isolates of *P. aeruginosa* and in response to exposure to the tryptophan catabolites. In general, IAA had a more expressive anti‐virulence effect in clinical isolates than indole or IA. Changes in the virulence of *P. aeruginosa* caused by IAA *in vitro* were also reproduced in a murine model of lung infection. Mice infected with PAO1 grown in the presence of IAA showed lower bacterial load in the lung and reduced inflammatory response at the site of infection, with reduced lethality rate. Altogether, these data suggest that IAA might represent an interesting scaffold for the development of anti‐virulence molecules targeting *P. aeruginosa*.

## 4 Methods

### 4.1 Strains and Growth Conditions

Laboratory strains of *Pseudomonas aeruginosa* (PAO1, PA14, PA103) and clinical isolates were cultured on Pseudomonas Isolation Agar (PIA) plates and incubated at 37°C for 24 h. A single colony was transferred to 40 mL of Luria–Bertani broth and incubated under shaking at 37°C for 16 h. For liquid culture experiments, bacterial suspensions were diluted to an OD600 of 0.1, with or without different tryptophan metabolites.

A total of 30 clinical isolates were obtained from patients admitted to the Intensive Care Unit of Hospital Risoleta Tolentino Neves (HRTN) in Belo Horizonte, Minas Gerais, between January 1 and December 31, 2019. Patients were aged 18 years or older, of both sexes, and met the hospital infection criteria established by the National Nosocomial Infection Surveillance System (NNISS). Pathogens were isolated and identified from microbiological analysis of tracheal aspirates, bronchoalveolar lavage, sputum, blood cultures, catheters, and nasal and perianal swabs, processed at the Clinical Pathology Laboratory of HRTN. Reference strains PAO1, PA103, and PA14 were used as controls. This study was approved by the hospital's Teaching, Research, and Extension Core (NEPE ‐ protocol 24/2018).

### 4.2 Preparation of Metabolites

Tryptophan metabolites obtained from SIGMA‐ALDRICH were diluted in 1% v/v dimethylformamide (DMF) and maintained at 4°C. For the experiments, indole, indoleacetic acid (IAA), indolealdehyde (IAld), indoxyl‐3‐sulfate (I3S), indoleacrylic acid (IA), and indole‐propionic acid (IPA) were diluted to 100 µM.

### 4.3 Growth Curve Analysis

The growth curve analysis was performed using PAO1 and PA14 strains. For this, 20 µL of the inoculum at 10^6^ CFU/mL was added to 180 µL of LB broth in a 96‐well plate. The plate was incubated at 37°C in a plate reader for 60 h. Every 1 h, the absorbance was read at 600 nm to create an absorbance versus time curve.

### 4.4 Crystal Violet Biofilm Assay

Overnight cultures (OD600:= 0.1) were incubated in polystyrene 96‐well plates at 37°C for 24 h with or without 100 µM tryptophan metabolites. After incubation, wells were washed with PBS, fixed with methanol, and stained with 0.5% crystal violet for 10 min. Excess dye was removed with sterile water, and biofilm was solubilized with ethanol‐acetone (4:1) for 10 min. The solution was transferred to a flat‐bottom 96‐well plate, and absorbance was measured at 620 nm (Cusumano et al. 2025).

### 4.5 Swimming, Swarming and Twitching Motility Measurement

Swimming motility was assessed using 0.7% agar with 1% tryptone and 0.25% NaCl, swarming motility with BM2 swarming medium, and twitching motility with LB agar (1.0%). Tryptophan metabolites (100 µM) dissolved in DMF were incorporated into the agar. Overnight cultures (OD600 = 1) were inoculated at the center of the agar, and halo diameters were measured after 24 h at 37°C.

### 4.6 Pyocyanin and Pyoverdine Quantification

*P. aeruginosa* strains were cultured in 5 mL of peptone broth with or without metabolites (100 µM) at 37°C for 48 h. After centrifugation (5000×*g*, 10 min), the supernatant was transferred to a tube with 3 mL of chloroform and centrifuged (1000×*g*, 5 min). The organic phase was mixed with 1 mL of 0.2 M HCl, and absorbance was measured at 530 nm. For pyoverdine quantification, strains were grown in 5 mL of peptone broth at 37°C for 24 h. After centrifugation (5000×*g*, 10 min), the supernatant's absorbance was measured at 404 nm (Chandler et al. 2019).

### 4.7 Sensitivity to Hydrogen Peroxide Assay

The sensitivity of *P. aeruginosa* strains to hydrogen peroxide was assessed using the agar diffusion method. Bacterial suspensions were uniformly inoculated onto Pseudomonas Isolation Agar plates. Wells (6 mm) were cut into the agar, and 50 µL of 30% hydrogen peroxide was added. After overnight incubation at 37°C, inhibition zone diameters were measured.

### 4.8 Enterobacterial Repetitive Intergenic Consensus (ERIC)‐PCR

ERIC sequences were amplified from DNA (50 ng) extracted from isolates and reference strains using universal primers for enterobacteria: Primer 1 (5’‐ATGTAAGCTCCTGGGGATTCAC‐3’) and Primer 2 (5’‐AAGTAAGTGACTGGGGTGAGCG‐3’). PCR reactions (20 µL) were prepared with the GoTaq® G2 Hot Start Taq Polymerase kit (Promega, USA), using 10 µL of DNA and 10 µL of mix. Cycling conditions were: 94°C for 3 min (initial denaturation), followed by 40 cycles of 94°C for 30 s, 52°C for 1 min, and 72°C for 2 min, with a final extension at 72°C for 6 min.

For agarose gel electrophoresis, 10 µL of PCR product was mixed with 1 µL of BlueJuice™ Gel Loading Buffer (10X) (Invitrogen, Thermo Fisher Scientific) and loaded onto a 2% agarose gel in 1X TAE buffer. A 100 bp DNA Ladder (Thermo Fisher Scientific) was used as a molecular marker. Electrophoresis was run at 60 mV for 2 h, followed by staining with GelRed™ for 20 min.

Gel images were acquired using the ChemiDoc XRS system and Image Lab 6.0.1 software (Bio‐Rad, USA) and processed with GelJ (Java 8, Oracle, USA). A dendrogram was constructed using Pearson correlation coefficients and clustering by the unweighted pair group method with arithmetic mean (UPGMA).

### 4.9 Phenotypic Grouping

The 30 clinical isolates and 3 reference strains (PAO1, PA14 and PA103) of *P. aeruginosa* were grouped based on the results obtained with phenotypic tests using hierarchical clustering with Euclidean distance and wing linkage by the dendextend R package, version 1.14.0 and the heatmap was designed with the heatmaply R package, version 1.2.1 (Galili 2015).

### 4.10 Pseudomonas Aeruginosa Lung Infection in Mice

Female C57BL/6 mice (8 weeks old) were intranasally inoculated with *P. aeruginosa* PAO1 (1×10⁷ CFU/40 µL) grown with IAA, IA, indole, or DMF (vehicle). Weight and survival were monitored for 5 days post‐infection.

In another experiment, mice were euthanized 24 h post‐infection for bacterial burden assessment in bronchoalveolar lavage (BAL) and lung tissues via CFU counts. Leukocytes were quantified using a modified Neubauer chamber after Turk's dye staining. Differential counts were performed on Cytospin‐prepared slides stained with the panoptic kit (RenyLab).

### 4.11 Catalase Activity Assay

*P. aeruginosa* PAO1 was grown in LB broth at 37°C with shaking for 16 h, with or without 100 µM IAA. Then, 1 mL of culture was sonicated (3 cycles, 30 s each) and centrifuged (3000 rpm, 10 min). For catalase activity, 50 µL of supernatant was mixed with 50 µL of 10 mM hydrogen peroxide in a 96‐well plate, and absorbance was measured at 240 nm after 5 min. Protein content in the supernatant was assessed using the Bradford assay, comparing absorbance at 595 nm to a BSA standard curve.

### 4.12 Gene Expression Analysis by RT‐qPCR

RT‐qPCR was used to analyze the expression of *fliA, phzA, phzS, pvdS, pilA*, and *pqsA* in *P. aeruginosa* PAO1. RNA was extracted from 10 mL of an 8 h PAO1 culture at 37°C, with or without the tested metabolite. After centrifugation, the pellet was resuspended in 750 µL of Trizol, followed by chloroform extraction. The aqueous phase was transferred to a tube with isopropanol, washed with ethanol, and dried. RNA was resuspended in water and converted to cDNA using the iScript cDNA Synthesis Kit (Bio‐Rad). The qPCR reactions (10 µL) contained 5 µL of Power SYBR Green PCR Master Mix 2X (Applied Biosystems, USA), 1 µL each of forward and reverse primers (5 µM), 1 µL of Milli‐Q water, and 2 µL of cDNA. Amplifications were performed on a StepOne PCR System (Applied Biosystems, USA) using SYBR Green detection. Primer sequences are listed in Table S1.

### 4.13 Statistical Analysis

A normality test was performed to verify whether the samples had a Gaussian distribution. Statistical comparisons between the various groups were performed using ANOVA “one way” followed by the Newman‐Keuls post‐test. For comparison between two groups, when necessary, the “Student's *t*” test was used. The lethality and weight curve for infection in a murine model was evaluated by log‐rank and two‐way ANOVA tests, respectively. Results are presented as mean±mean standard error. The significance level adopted was *p* < 0.05. To carry out all the analyses, the GraphPad PRISM software, GraphPad software Inc. version 8 was used (San Diego, CA, USA).

## Author Contributions

**Carlos E. D. Igidio:** conceptualization, investigation, draft writing, methodology implementation, data visualization and formal analysis. **Camila B. Brito:** investigation, methodology implementation. **Rafael O. Bezerra:** investigation, methodology implementation. **Samantha N Oliveira:** investigation, methodology implementation. **Cinthia F Teixeira:** investigation, methodology implementation. **Barbara M Amorim‐Santos:** investigation, methodology implementation. **Allanis C O Andrade:** investigation, methodology implementation. **Deigo L Rios:** investigation, methodology implementation, data visualization and formal analysis. **Silvia H S P Pedroso:** investigation, methodology implementation. **Simone G dos Santos:** resources. **Mauro M Teixeira:** funding acquisition, resources, project administration. **Daniele G Souza:** funding acquisition, resources, project administration. **Camila P S M Mata:** resources, supervision. **Caio T. Fagundes:** conceptualization, investigation, funding acquisition, draft writing, data visualization and formal analysis, resources, supervision, project administration.

## Ethics Statement

The authors have nothing to report.

## Conflicts of Interest

The authors declare no conflicts of interest.

## Supporting information


**Figure S1:** Expression profile of virulence‐related parameters of Pseudomonas aeruginosa clinical isolates. **Table S1:** Primers used for RT‐qPCR.
