# Supplementary material for: Indole‐Acetic Acid Impairs Pseudomonas aeruginosa Virulence and Alters Lung Infection in Mice
Source: Microbiologyopen. 2025 Dec 12;14(6):e70185. doi: 10.1002/mbo3.70185 (PMC12699321; doi:10.1002/mbo3.70185)
Supplement: Supplementary file 1 — Figure S1: Expression profile of virulence‐related parameters of Pseudomonas aeruginosa clinical isolates. Table S1: Primers used for RT‐qPCR. [file MBO3-14-e70185-s001.pdf]

Supporting information

Indole-acetic acid impairs *Pseudomonas aeruginosa* virulence and alters lung infection in mice

Carlos Eduardo Dias Igídio[1,2], Camila Bernardo Brito[2], Rafael de Oliveira Bezerra[2], Samantha Neves Oliveira[1,2], Cinthia Firmo Teixeira[1,2], Bárbara Maria Amorim dos Santos[2], Allanis Cristiny Oliveira Andrade[1,2], Diego Lisboa Rios[2], Silvia Helena Sousa Pietra Pedroso[3], Simone Gonçalves dos Santos[3], Mauro Martins Teixeira[1], Daniele da Glória de Souza[2], Camila Pacheco Silveira Martins da Mata[4], Caio Tavares Fagundes[1,2*]

[1]Centro de Pesquisa e Desenvolvimento de Fármacos, Instituto de Ciências Biológicas, Universidade Federal de Minas Gerais, 31270901, Belo Horizonte, MG, Brazil.

[2]Laboratório de Interação Microrganismo-Hospedeiro, Departamento de Microbiologia, Instituto de Ciências Biológicas, Universidade Federal de MG, 31270901, Belo Horizonte, MG, Brazil.

[3] Laboratório de Microbiologia Oral e de Anaeróbios, Departamento de Microbiologia, Instituto de Ciências Biológicas, Universidade Federal de MG, 31270901, Belo Horizonte, MG, Brazil.

[4] Hospital Risoleta Tolentino Neves, 31744012, Belo Horizonte, MG, Brazil.

*Corresponding author email: caio.fagundes@gmail.com

SUPPORTING INFORMATION IN THIS DOCUMENT:

Supporting table: ST1

Supporting figure: S1

Table ST1: Primers used for RT-qPCR.

Gene	**Orientation**	**Sequence 5’-3’**
fliA	Forward	CTCCAATTGAGCCTCGAAGA
	Reverse	TTCGTTGTGACTGAGGCTGG
phzA	Forward	AACGGTCAGCGGTACAGGGAAAC
	Reverse	ACGAACAGGCTGTGCCGCTGTAAC
phzS	Forward	CAACGCCTATCCGCAGTACT
	Reverse	CCATCGGGTACTGCAGGATC
pvdS	Forward	TAACCGTACGATCCTGGTGAAGA
	Reverse	ACGATCTGGAACAGGTAGCTGAG
pqsA	Forward	GACCGGCTGTATTCGATTC
	Reverse	GCTGAACCAGGGAAAGAAC

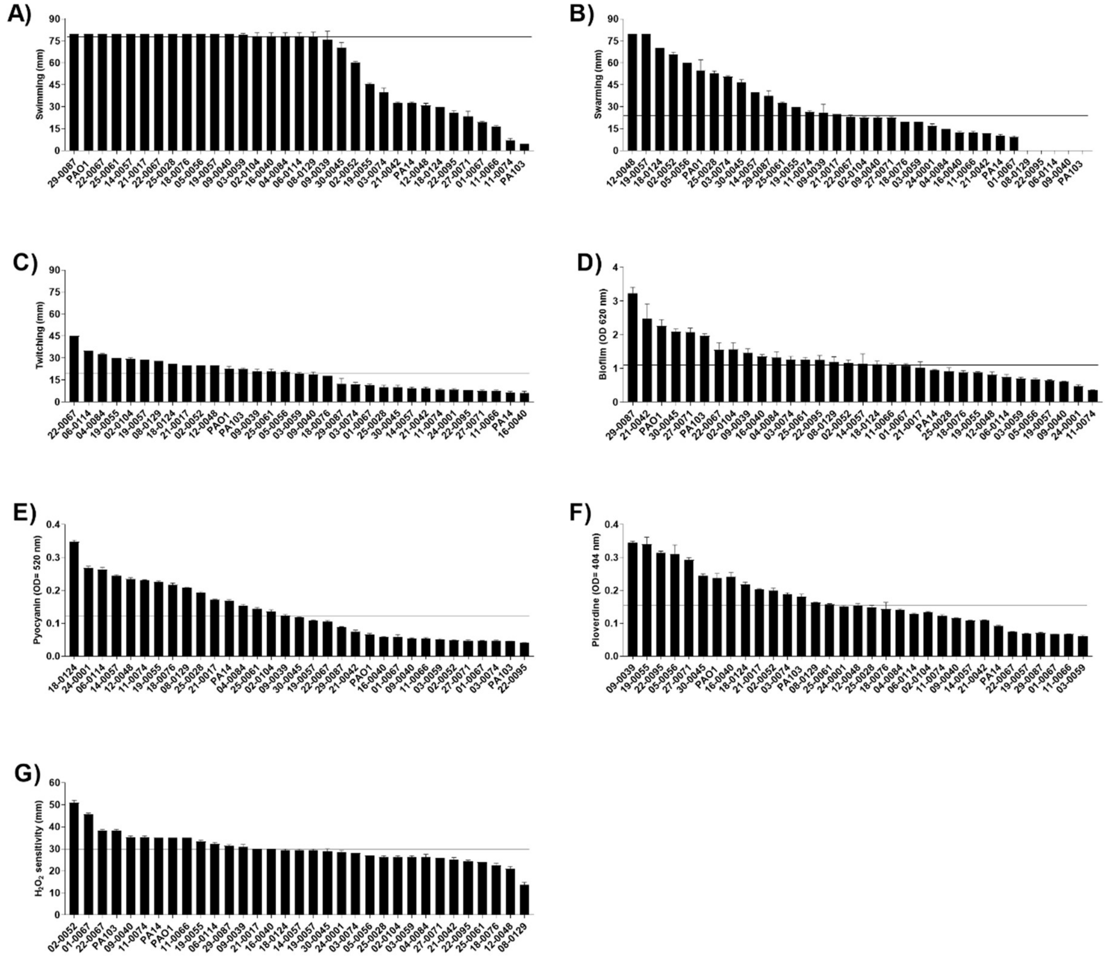

Figure S1: Expression profile of virulence-related parameters of *Pseudomonas aeruginosa* clinical isolates. All clinical isolates used in this study and the reference strains PAO1, PA14, and PA103 were subjected to swimming (A), swarming (B), twitching (C), biofilm (D), pyocyanin (E), pyoverdine (F) and sensitivity to H_2O_2 (G) tests to define the virulence profile for each sample. The samples are arranged in descending order of expression of each parameter. The line drawn over each graph represents the average of the values between the isolates. (A) Swimming motility, expressed as mm of motility halo; (B) Swarming motility, expressed as mm of motility halo; (C) Twitching motility, expressed as mm of motility halo; (D) Biofilm matrix production, as expressed as O.D. of crystal violet dye incorporation; (E) Pyocyanin production, expressed as O.D. in the extract from culture supernatants; (F) Pyoverdine production, expressed as O.D. in the extract from culture supernatants; G) Hydrogen peroxide sensitivity, expressed as mm of inhibition halo. N=3 per strain.
